# Comparison of Fecal Microbial Composition and Antibiotic Resistance Genes from Swine, Farm Workers and the Surrounding Villagers

**DOI:** 10.1038/s41598-017-04672-y

**Published:** 2017-07-10

**Authors:** Jian Sun, Ting Huang, Chong Chen, Ting-Ting Cao, Ke Cheng, Xiao-Ping Liao, Ya-Hong Liu

**Affiliations:** 1 National Risk Assessment Laboratory for Antimicrobial Resistance of Animal Original Bacteria, South China Agricultural University, Guangzhou, China; 2 Guangdong Provincial Key Laboratory of Veterinary Pharmaceutics Development and Safety Evaluation, South China Agricultural University, Guangzhou, China

## Abstract

The external environment plays a critical role in shaping the structure of the gut microbiome. One potential health threat lies in the release of antibiotic resistant genes (ARGs) from cross-contaminated microbiomes. We focused this study on a comparison of fecal microbial composition and antibiotic resistant genes between farm workers, local villagers and swine. We used a high-throughput next-generation sequencing of 16S rRNA and real-time PCR for these studies. Our results indicated that workers had less species diversity as compared to the local villagers. Moreover, the bacterial communities of the farm workers, the local villagers and swine feces were clearly divided into three groups. The workers had a greater abundance of *Proteobacteria* as compared to swine and the local villagers. The *Clostridiaceae* in the workers and swine were more abundant than the local villagers. In addition, there were ARG differences between the farm workers or local villager’s and swine feces. The farm workers and the local villagers had similar relative abundance except for macrolide ARGs. Taken together, these data suggest that the swine farm environment affects the fecal bacterial composition of swine farm workers. However, ARG spread was influenced by factors independent of the swine farm environment.

## Introduction

Bacteria in the mammalian gastrointestinal tract play an important role in human health^1–3^. This includes altering metabolic capabilities^4, 5^, providing pathogen protection and immune system programming^4, 6–8^. In addition, external factors such as diet and environment play a critical role in shaping the composition and structure of the gut microbiome^9–11^. Alterations in these factors can change the gut biodiversity; a high biodiversity is associated with health while a low biodiversity is linked to pathological states^12^. In adults, the majority of bacteria found in the gut belong to the bacterial phyla *Bacteroidetes* and *Firmicutes*. The *Actinobacteria*, *Fusobacteria* and *Verrucomicrobia* occur less frequently and their levels can vary widely among individuals^13–17^.

A shared environment, such as contact with same microbial sources, also results in similar fecal microbiota^18–20^. Microbe exchange between individuals is facilitated by direct contact and an individual’s microbial community is shaped by their surroundings including animals^21^. For instance, a swine farm environment most likely influences farm workers gut microbial communities since the workers spend the bulk of their time in contact with the animals or the farm environment.

The emergence of resistance to multiple antimicrobials among both animal and human pathogens is rapid and widespread^22^. Antimicrobial-resistant bacterial fecal populations pose a potential public health threat due to their environmental release^23^. Importantly, mobile genetic elements harboring antibiotic resistance genes can also transfer between different gut microbiota species. There is substantial evidence indicating a risk of animal-to-human transmission of resistant bacteria^24^. There are a wide range of estimates on the prevalence of resistant bacteria, such as methicillin-resistant *Staphylococcus aureus* (MRSA), multidrug-resistant *Staphylococcus aureus*, extended-spectrum beta-lactamase (ESBL) and plasmid-mediated AmpC beta-lactamase-producing *Enterobacteriaceae* in people in contact with livestock. This includes pig farms, veterinarians and slaughterhouse workers from many countries^25–28^. Therefore, to determine the impact of the swine farm environment among farm workers necessitates determining the paths of antibiotic resistant gene (ARG) transfer.

In this study, we compared the influence of swine farm environment on the fecal communities and antibiotic resistant genes among the farm workers, local villagers and swine in Southern China.

## Results

### Bacterial species distributions and diversity

The diversity estimated of the samples were calculated after quality control (Table S1).We used the Simpson, Shannon, abundance-based coverage (ACE), observed species and Chao indices as a measure of the alpha diversity (evenness and richness) of the microbial communities. A higher Simpson index indicates less species evenness^29^.

**Figure 1:**
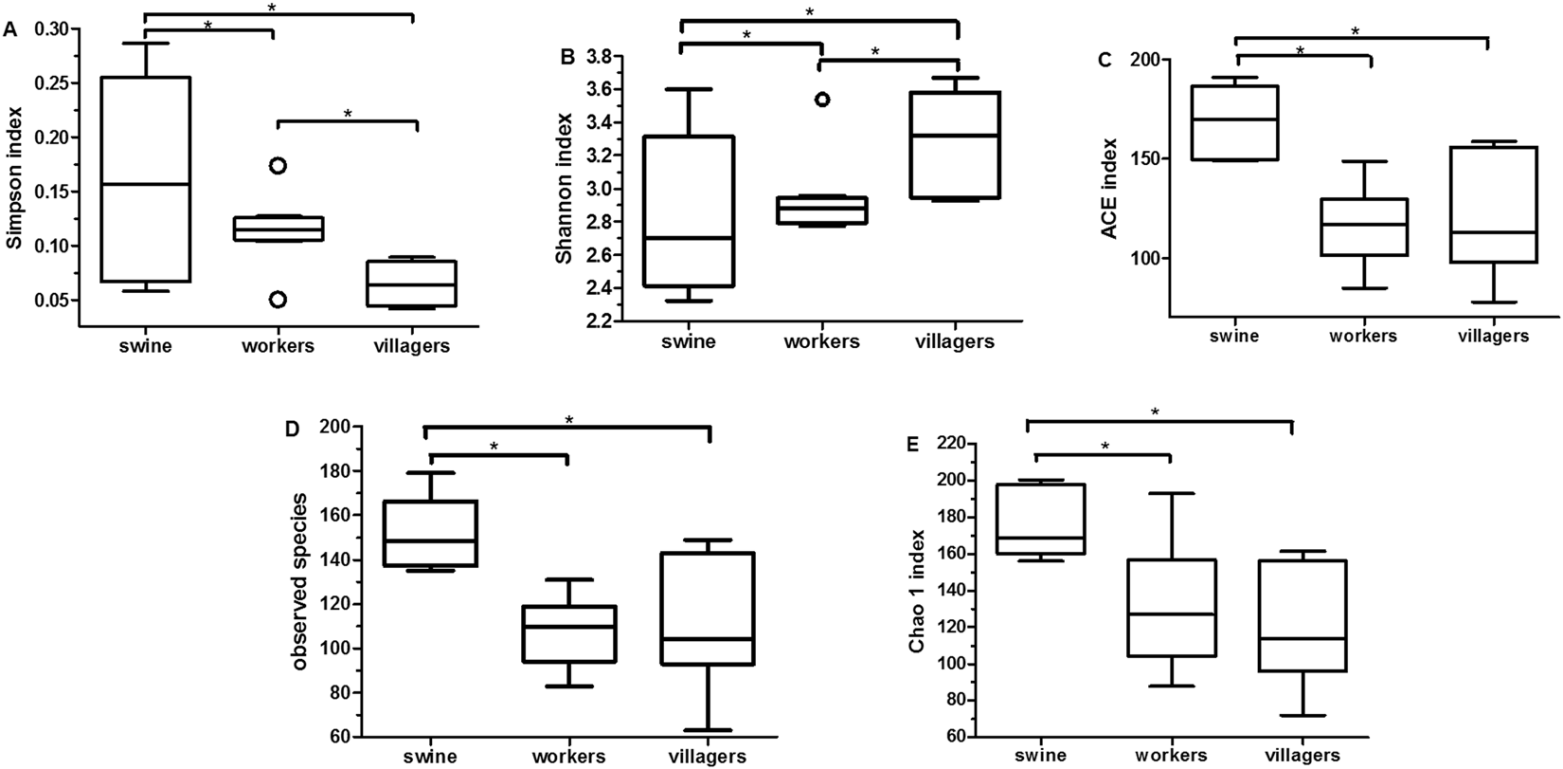
Box plots of diversity and richness estimators. Panel (A): Simpson index; Panel (B): Shannon index; Panel (C): Abundance-based coverage (ACE); Panel (D): Observed species; and Panel (E): Chao 1 index. “O” indicates outlier values, *p < 0.05.

Farm workers had significantly less species diversity as compared with local villagers (Fig. 1A and B). There were no significant differences between the farm workers and the local villagers regarding the richness of the communities (Fig. 1C, D and E). Swine had the lowest bacterial species diversity (Fig. 1A and B), but had the highest species richness as compared to farm workers and local villagers (Fig. 1C, D, and E).

### Bacterial species clusters

**Figure 2:**
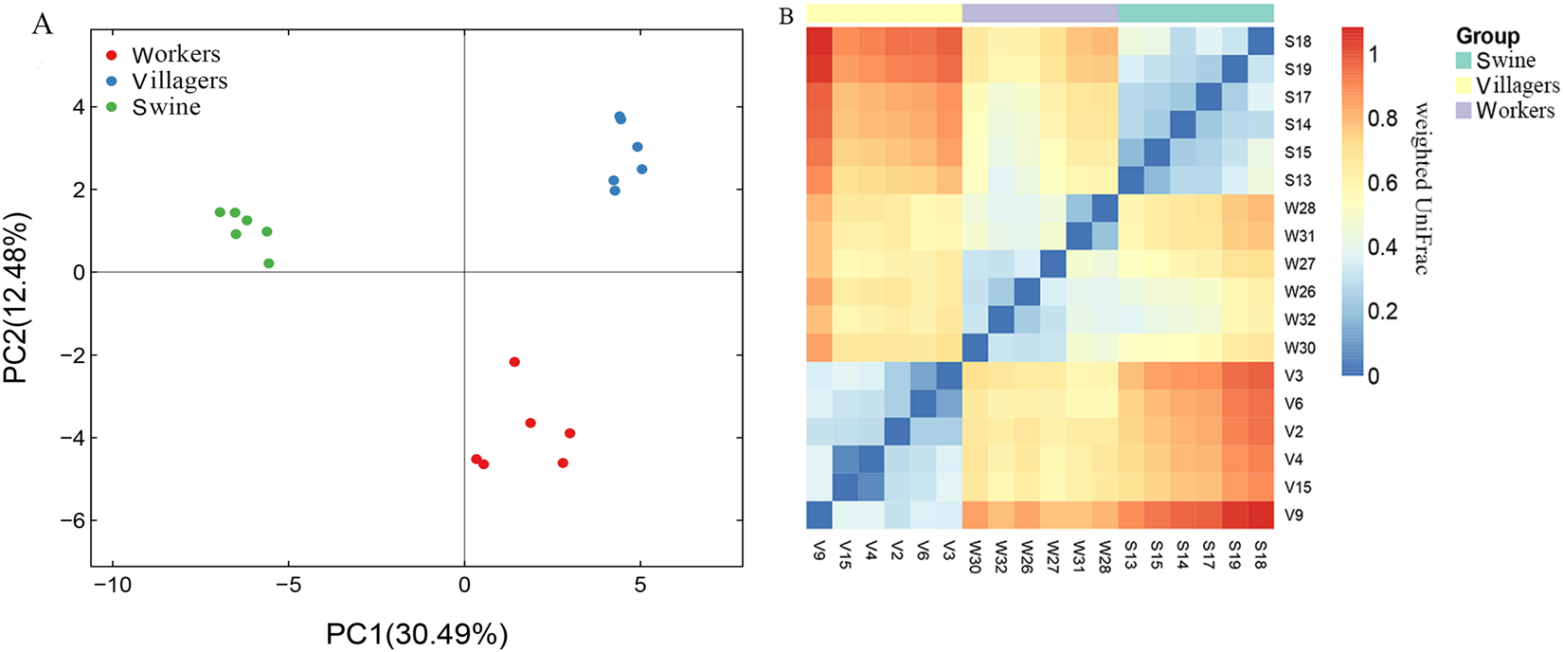
Principal components analysis (PCA) and β diversity. Panel (A): PCA plot describing the dissimilarity between three different groups. Colored markers are used to differentiate samples. Panel (B): Heatmap of diversity distance calculated by weighted Unifrac. The distance index ranges from absent (blue) to abundant (red).

The bacterial communities of the farm workers, the local villagers and swine feces were clearly divided into three groups according to principal component analysis (PCA) (Fig. 2A). The heatmap of weighted UniFrac diversity distance analysis and weighted and unweighted UniFrac cluster tree, indicated that when farm workers and local villagers were compared with swine, the bacterial communities in feces of the farm workers were more similar with the swine feces (Figs 2B and S1).

### Bacterial structure

**Figure 3:**
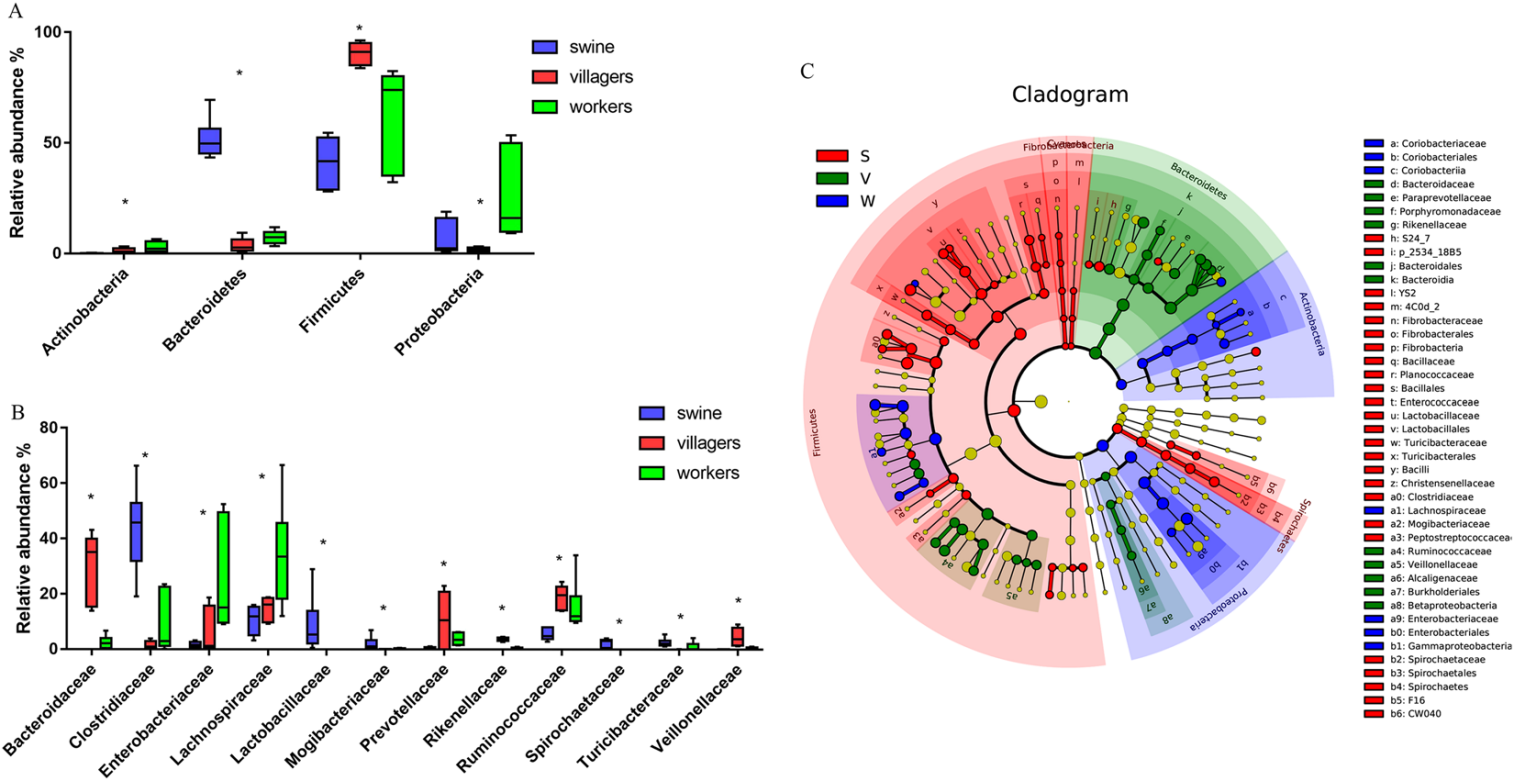
Relative abundance of bacterial composition. Panel (A), microbial phyla. Panel (B), families. *p < 0.05, Kruskal-Wallis test. Only taxa with a mean relative abundance of ≥1% are shown. Panel (C). LEfSe among different groups. Small circles with different colors in the diagram represent abundance of those taxa in the respective group. Yellow circles represent non-significant differences in abundance among three different groups of those particular taxa. The brightness of each dot is proportional to its effect size.

At the phylum level, the farm workers and swine had significantly higher and lower relative abundances of *Bacteroidetes* and *Firmicutes*, respectively, as compared to the local villagers (Fig. 3A). Moreover, the workers had a greater abundance of *Proteobacteria* as compared to the swine and the local villagers. At the family level, the *Clostridiaceae* were more abundant in the feces of farm workers and swine compared with the local villagers. In addition, the *Enterobacteriaceae* and *Lachnospiraceae* in the workers feces were more abundance than those from the local villagers (Fig. 3B).

LEfSe analysis for metagenomic biomarker tracing between microbial communities indicated that the *Coriobacteriaceae*, *Lachnospiraceae* and *Enterobacteriaceae* were enriched in the farm workers feces. Conversely, feces from the local villagers were enriched for Bacteroidaceae, *Paraprevotellaceae*, *Porphyromonadaceae*, *Rikenellaceae*, *Ruminococcaceae*, *Veillonellaceae* and *Alcaligenaceae* (Fig. 3C).

### Occurrence of antibiotic resistance genes

**Figure 4:**
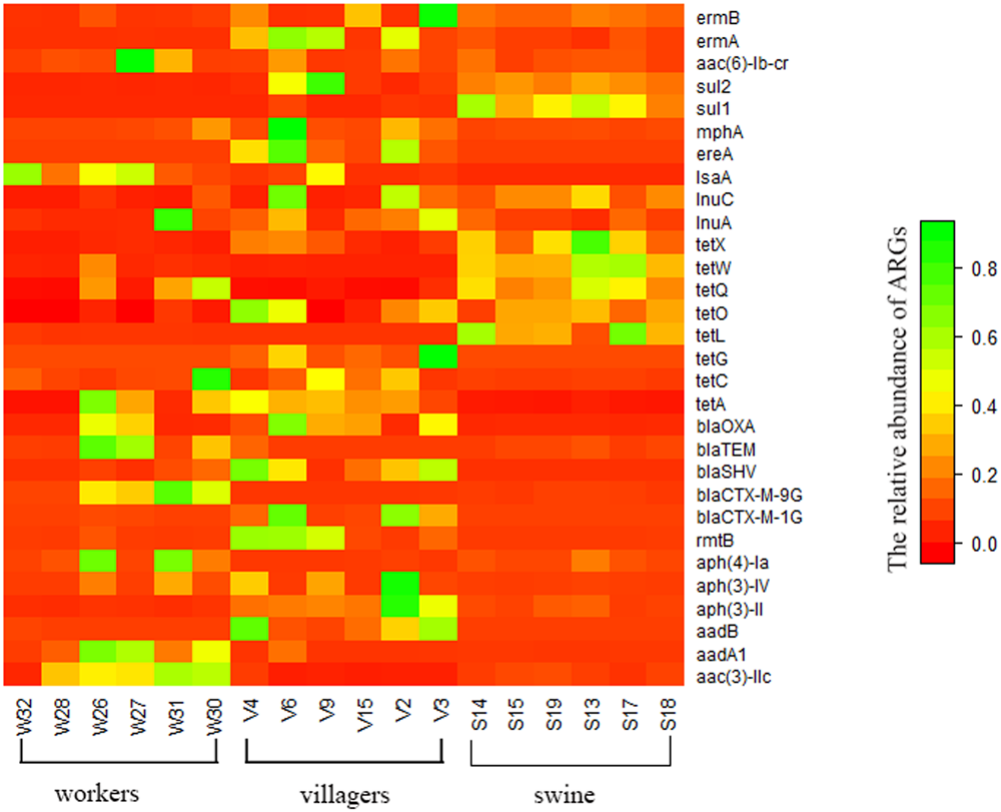
Relative abundances of ARGs among the swine, farm workers and local villagers. The relative abundance of each ARG ranges from absent (red) to abundant (green).

When we measured ARG distributions between swine feces and the human samples, farm workers and villagers’ feces possessed multiple ARGs. In contrast, the swine feces contained ARGs that were specific for tetracycline, lincomycin and sulfanilamide. However, farm workers and villagers had similar relative ARG levels except for the macrolides (Fig. 4 and Table 1). A comparison of farm workers and villagers versus swine indicated there were significant differences in the ARG distribution among all the antibiotic classes studied, except for the lincomycin and macrolide classes (Fig. 4 and Table 1).

**Table 1:** Relative abundance of ARGs in swine, farm workers and local villagers. All data were normalized to ambient 16S rRNA copies.

ARGs	workers	villagers	*P* ^a^	swine	*P* ^b^
aminoglycoside inactivating enzymes	4.49E-01 ± 5.03E-02	5.68E-01 ± 7.54E-02	0.531	6.69E-02 ± 3.82E-03	0.003
β-lactamases	1.90E-01 ± 4.80E-02	4.46E-01 ± 7.64E-02	0.119	5.59E-03 ± 9.27E-04	0.017
tetracycline	5.90E-01 ± 8.43E-02	5.45E-01 ± 4.19E-02	0.860	1.20E + 00 ± 5.43E-02	0.008
lincomycin	2.20E-01 ± 5.43E-02	6.84E-02 ± 3.49E-02	0.762	1.88E-03 ± 4.62E-04	0.312
macrolide	3.74E-04 ± 3.11E-04	1.68E-01 ± 7.94E-02	0.040	8.11E-05 ± 2.83E-05	0.184
sulfanilamide	1.70E-04 ± 1.63E-04	4.61E-02 ± 3.11E-02	0.122	7.43E-02 ± 1.00E-02	0.031
PMQR	2.02E-03 ± 3.07E-03	5.80E-04 ± 7.03E-04	0.330	4.99E-04 ± 3.48E-04	0.442
MLSB	5.44E-03 ± 3.40E-03	2.74E-01 ± 1.16E-01	0.314	5.27E-02 ± 1.12E-02	0.010

^a^Indicated the *P* values between the workers and villagers. ^b^Indicated the *P* values between the human (workers and villagers) and swine.

## Discussion

In this investigation, we found that farm workers had significantly less species diversity in their fecal bacteria compared to the local villagers (Fig. 1). We could also separate the structure of the workers fecal bacteria from the local villager feces (Fig. 2A). The composition of the farm workers fecal microbiota was dominated by *Firmicutes* and *Proteobacteria*, while the local villagers were dominated by the *Firmicutes and Bacteroidetes* (Fig. 3A). Macrolide ARG levels in the local villagers were greater than farm workers, and the swine had the lowest abundance (Fig. 4 and Table 1).

Microbiome compositions change drastically during the early years of life and remain stable thereafter in the gut^30^. The core functions of the gut include glycosaminoglycan biodegradation, the production of several short-chain fatty acids, enrichment for specific lipopolysaccharides and the production of vitamins and essential amino acids^31–34^. Under long-term selective pressures (such as diet), a given population tends to enrich for some of these functions^35^ and a high diversity is generally associated with good health^36^. Less diversity or evenness in a bacterial community diminishes its ability to withstand perturbations^37^. Even though hosts with less diverse bacterial communities may not exhibit overt disease, these communities are less than optimal for preventing disease. These individuals may be more susceptible obesity^38^, inflammatory bowel disease^39^ and types 1 and 2 diabetes^30, 40^. Moreover, this loss of diversity may be linked to a high fat, high-refined-sugar and low-fiber diet^31^. In our current study, we demonstrated that the workers had less species diversity compared with the villagers, suggesting the farm workers have a higher risk to their health.

*Firmicutes* and *Proteobacteria* dominated farm workers feces instead of *Bacteroidetes*. The gut microbiota constitutes the most abundant microbial domain within the human body with the vast majority belonging to bacteria phyla *Firmicutes* and *Bacteroidetes* ^41^. At the phylum level, a higher abundance of *Bacteroidetes* and *Firmicutes* were observed in uninfected family members compared to hospital patients, whereas *Proteobacteria* were more abundant in patients^42^. A relatively higher proportion of *Proteobacteria* in the farm workers was detected in our current study. Enhanced levels of *Proteobacteria* may be indicative of a diseased state that commonly occurs during enteric infection or following perturbation of the microbiota^42^.

ARGs are becoming recognized as contaminants independent of their bacterial host^43^. To pose a human health risk, agricultural ARGs must be in or transferred to a human pathogen with which humans have direct physical contact^44^. Many studies have indicated that ARG transmission from antibiotic resistant bacteria is possible between animals and humans^25–28^. However, in the current study, we found large differences in ARG levels between swine and farm workers or villagers’ feces. The relative ARG abundance in farm workers were not significantly higher than villagers, except for the macrolides. This suggests that the swine farm workers occupational exposure to the swine farm environment does not appear to be associated with the prevalence of genotype resistance in the swine. This finding agrees with that of a previous study^45^.

These results also indicated that farm workers and swine had divergent gut bacterial compositions. Many swine bacterial species cannot survive in the human gut. Therefore, resistant bacteria in the swine gut would not colonize the farm workers. Moreover, the farm workers were more mobile and had a more diverse diet and therefore, more opportunities to acquire resistant bacteria. Therefore, the spread of ARGs in swine farm workers may be influenced by more than the swine farm environment.

In conclusion, we demonstrated that swine farm workers living in the swine farm environment have a higher risk of dysbiosis in the gut microbiome that might influence their health. However, ARG spread in swine farm workers may be influenced by more than the swine farm environment.

## Materials and Methods

### Fecal sample collections

Fresh fecal samples were collected randomly from six swine farm workers (W group, 3 males and 3 females, aged 36–45 years) who had been working for more than one year in a swine farm located in Huizhou in Guangdong province, China. The control group consisted of six local villagers (V group, 3 males and 3 females, aged 29–42 years) who have been living in the Huizhou and removed from any livestock farm for more than 10 years. The workers and local villagers were in good health, with no chronic metabolic diseases and had not received any antibiotics for at least one month prior to study. Fecal samples were also collected from six swine (S group, 3 males and 3 females, aged 1 year’s old, administrated tetracycline and sulfonamide antibiotics) that had been in contact with the workers. The samples were frozen on dry ice at the point of collection and stored at −80 °Cuntil analysis.

This study protocol was reviewed and approved by the South China Agriculture University Animal ethics committee. All individuals before participation indicated informed consent. The owners of the farm swine from which feces were taken gave permission for their swine to be used in this study.

### DNA extraction

Fecal DNA was extracted using QIAamp Fast DNA Stool Mini Kit (Qiagen, Valencia, CA, USA) according to the manufacturer’s instructions. Total DNA was quantified using a NanoDrop ND-2000 UV spectrophotometer (NanoDrop Technologies, Wilmington, DE). Only DNA samples with A260/A280 > 1.7 and A260/A230 > 1.8 were used for further analysis^46^. The extracts were stored at −20 °C until use^42^.

### 16S rRNA gene sequencing

The V3 + V4 hypervariable regions of 16S rDNA were amplified from microbial genomic DNA harvested from fecal samples by PCR. PCR primers flanking the V3 + V4 hypervariable region of bacterial 16S rDNA were designed. The barcoded fusion forward primer (341 F 5′-CCTACGGGNGGCWGCAG-3′), and a reverse primer (805 R 5′-GACTACHVGGGTATCTAATCC-3′) were used as previously described^47, 48^. The PCR conditions were as follows: one pre-denaturation cycle at 94 °C for 4 min, 25 cycles of denaturation at 94 °C for 4 min 30 s, annealing at 55 °C for 45 s, and elongation at 72 °C for 30 s, and one post-elongation cycle at 72 °C for 5 min. The PCR amplicons were separated by electrophoresis in 0.8% agarose gels, and single bands were extracted from the gels. Only PCR products without primer dimers and contaminant bands were used for DNA sequencing. Barcoded V3 and V4 amplicons were sequenced by Illumina MiSeq with a 7-cycle index read. Sequences with an average Phred (Q) score lower than 30, ambiguous bases, homopolymer runs exceeding 6 bp, primer mismatches, or sequence lengths shorter than 100 bp were removed. Only sequences with an overlap longer than 10 bp and without any mismatches were assembled according to their overlap sequence. Reads that could not be assembled were discarded. Barcode and sequencing primers were trimmed from the assembled sequences^49^.

### Sequencing data analysis

Sequencing data were analyzed using the Mothur software package v.1.33.0^50^. DNA sequences were aligned using CustalW (http://www.ebi.ac.uk/Tools/msa/clustalw2/) and trimmed to remove non-overlapping ends. Bacterial sequence reads were compared to a reference database of known 16S rRNA genes using the Ribosomal Database Project (RDP) databases (http://rdp.cme.msu.edu/seqmatch/seqmatch_intro.jsp). Taxonomic assignments were based on RDP classifiers^51^. The bacterial diversity of microbial communities was calculated using sequences from 6 samples per group with an Operational Taxonomic Unit (OTU) defined at an identity cut-off of 97%^52^. Alpha diversity index estimators were determined using the Mothur package^50^. A 3% dissimilarity level between sequences was used to calculate the diversity estimators. The microbial community structures in different samples were compared using the weighted UniFrac^53^. Principal component analysis (PCA) was conducted to assess the relationships among different groups. Heatmap figures were created using the R package. Linear discriminant analysis effect size was used to identify differences in taxa composition. Linear discriminant analysis effect size (LEfSe) was performed using the LEfSe web tool on taxonomic assignments from RDP’s sequence classifier^54, 55^. The LEfSe program was used to identify indicator organisms most likely to explain the differences among different groups with a logarithmic cutoff value of linear discriminant analysis (LDA) > 0.4.

### ARG Detection

PCR assays were used to detect eight well characterized ARG types^56^: plasmid-mediated quinolone resistance (PMQR) (*qnrA*, *qnrB*, *qnrC*, *qnrD*, *qnrS*, *aac(6*′*)-Ib-cr*, *qepA* and *oqxA*), tetracycline (*tet*(A), *tet*(C), *tet*(G), *tet*(L), *tet*(M), *tet*(O), *tet*(Q), *tet*(W) and *tet*(X)), macrolide (*mefA*, *msrA*, *msrD*, *ereA*, *ereB*, *mphA* and *mphC*), lincomycin (*lnuA*, *lnuB*, *lnuC*, *lnuD*, *lnuF*, *lsaA*, *lsaB* and *lsaC*), aminoglycoside inactivating enzymes (*aac(3*′*)-Ia*, *aac(3*′*)-IIc*, *aadA1*, *aadB*, *aph(3*′*)-II*, *aph(3*′*)-IV*, *aph(4*′*)-Ia*, *armA*, *rmtA*, *rmtB*, *rmtC*, *rmtD* and *aac(6*′*)-Ib*), β-lactamases (*bla*_TEM_, *bla*_SHV_, *bla*_DHA_, *bla*_CTX-M-1G_, *bla*_CTX-M-2G_, *bla*_CTX-M-9G_, *bla*_CTX-M-25G_, *bla*_CMY-2_ and *bla*_OXA_), sulfanilamide (*sul1*and *sul2*) and macrolide, lincosamide and streptogramins B (MLSB) (*ermA*, *ermB*, *ermC* and *ermTR*) in the fecal samples. All primers used in this study were based on previous studies^56, 57^. To ensure reproducibility, three replicates for each sample were performed in each run. The amplicons were visualized using electrophoresis through 1% agarose gels and detected using GelGreen Nucleic Acid gel stain (Biotium, Hayward, CA, USA). ARGs detected by PCR as well as the 16S rRNA genes were further quantified by qPCR using SYBR Premix Ex Taq (TAKARA Bio, Otsushi, Japan) in a Bio-Rad iQ5 thermal cycler (Hercules, CA, USA) according to the manufacturer’s instructions. Cycling conditions were as follows: 94 °C for 5 min followed by 35 cycles at 94 °C for 1 min, 60 °C for 1 min and 72 °C for 1 min with a final extension at 72 °C for 5 min. Melting curve analysis was performed for every assay from 60 to 95 °C with 1 °C intervals. A Eubacterial 16S rRNA gene was also included so that ARG levels could be normalized to the total bacterial community. This step provided a means to correct for potential variations in extraction efficiencies and to compare ARGs proportionally between samples of different overall population sizes. The ARG copy numbers were normalized to 16S rRNA represented in the sample that was used as the control. All experiments were performed in triplicate and the standard error of the measurements was determined from these parallel data. The different groups were analyzed by one-way ANOVA using SPASS 19.0.

### Statistical analysis

Relative ARG abundance among different groups were analyzed by ANOVA/LSD post hoc testing using SPSS 19.0. Differential abundance of bacterial taxa among three groups were compared using Fisher’s exact test at a statistical difference level of *p* < 0.05^58–61^.

## Electronic supplementary material

Supplementary Information
