# Supplementary material for: Comparison of Fecal Microbial Composition and Antibiotic Resistance Genes from Swine, Farm Workers and the Surrounding Villagers
Source: Sci Rep. 2017 Jul 10;7:4965. doi: 10.1038/s41598-017-04672-y (PMC5503985; doi:10.1038/s41598-017-04672-y)
Supplement: Supplementary file 1 — Supplementary Information [file 41598_2017_4672_MOESM1_ESM.doc]

**Comparison of Fecal Microbial Composition and Antibiotic Resistance Genes from Swine, Farm Workers and the Surrounding Villagers**

Jian Sun^1,2,*^, Ting Huang^1,2,*^, Chong Chen^1,2^, Ting-Ting Cao^1,2^, Ke Cheng^1,2^, Xiao-Ping Liao^1,2^, Ya-Hong Liu^1,2^

^1^National Risk Assessment Laboratory for Antimicrobial Resistance of Animal Original Bacteria, South China Agricultural University, Guangzhou, China

^2^Guangdong Provincial Key Laboratory of Veterinary Pharmaceutics Development and Safety Evaluation, South China Agricultural University, Guangzhou, China

^*^ These authors contributed equally to this work. Correspondence and requests for materials should be addressed to Ya-Hong Liu (email: lyh@scau.edu.cn).

Table S1. Summary of the 16S rRNA gene sequencing data.

| Group | Sample | Clean data (Mbp) | Connect tag number | OTU number |
| --- | --- | --- | --- | --- |
| Swine | S13 | 9.47 | 16086 | 138 |
|  | S14 | 10.02 | 17001 | 135 |
|  | S15 | 10.37 | 17622 | 179 |
|  | S17 | 8.33 | 14145 | 162 |
|  | S18 | 8.62 | 14620 | 146 |
|  | S19 | 10.38 | 17626 | 151 |
| Villagers | V15 | 10.44 | 17858 | 149 |
|  | V2 | 10.47 | 18052 | 103 |
|  | V3 | 10.29 | 17426 | 103 |
|  | V4 | 10.30 | 16857 | 141 |
|  | V6 | 10.46 | 18373 | 105 |
|  | V9 | 10.49 | 14506 | 63 |
| Workers | W26 | 7.85 | 13349 | 98 |
|  | W27 | 10.50 | 17869 | 105 |
|  | W28 | 8.60 | 14617 | 83 |
|  | W30 | 10.49 | 17912 | 131 |
|  | W31 | 7.38 | 12535 | 114 |
|  | W32 | 10.49 | 17921 | 115 |


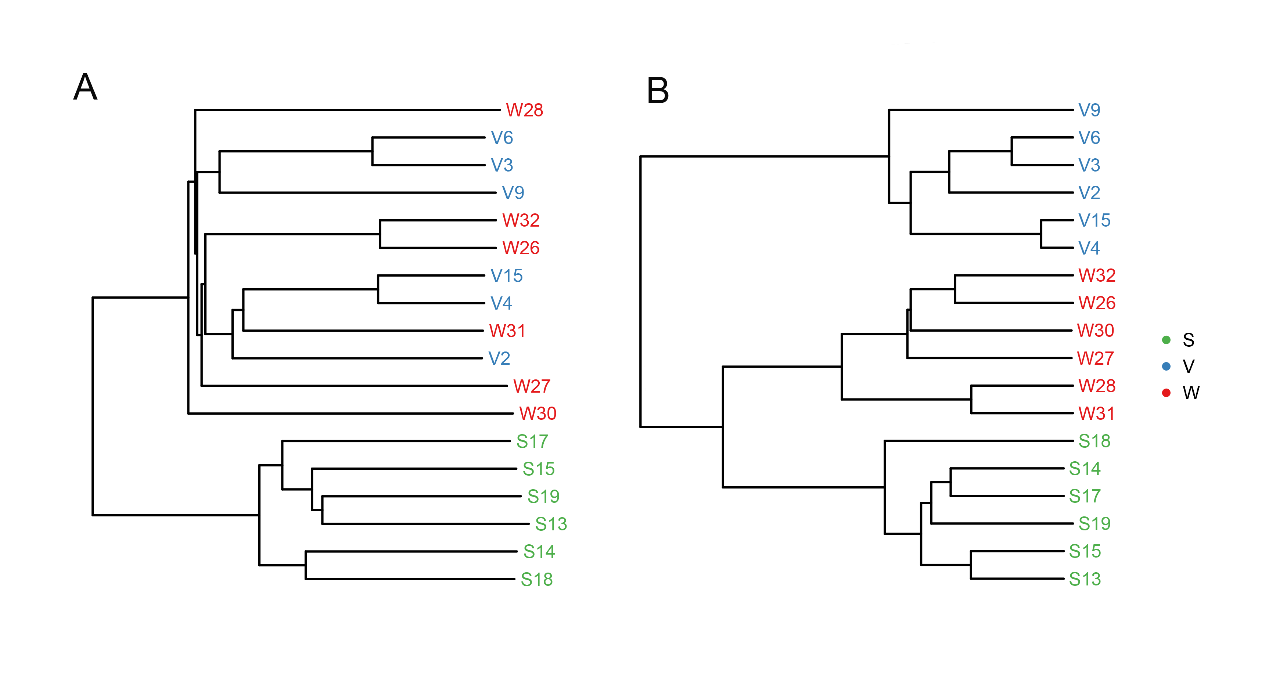


Fig S1 UniFrac cluster tree. Panel A for unweighted UniFrac cluster tree; Panel B for weighted UniFrac cluster tree. (Green for swine group; blue for villager group; red for worker group)
